# Lipidomic and metabolomic changes in community-acquired and COVID-19 pneumonia

**DOI:** 10.1016/j.jlr.2024.100622

**Published:** 2024-08-21

**Authors:** Mireia Saballs, Sandra Parra, Neus Martínez, Nuria Amigo, Lydia Cabau, Simona Iftimie, Raul Pavon, Xavi Gabaldó, Xavier Correig, Silvia Paredes, Josep Maria Vallvé, Antoni Castro

**Affiliations:** 1 Internal Medicine Department, Quiron Salud Hospital, Barcelona, Spain; 2 Research Group of Autoimmunity, Infection and Thrombosis (GRAIIT), Pere Virgili for Health Research Institute (IISPV), Rovira and Virgili University (URV), Reus, Spain; 3 Internal Medicine Department, “Sant Joan” University Hospital, Reus, Spain; 4 Biosfer Teslab, Reus, Spain; 5 Department of Basic Medical Sciences, Rovira and Virgili University (URV), Pere Virgili for Health Research Institute (IISPV), Tarragona, Spain; 6 Centre for Biomedical Research Network on Diabetes and Associated Metabolic Diseases (CIBERDEM), ISCIII, Madrid, Spain; 7 Clinical laboratory Department, “Sant Joan” University Hospital, Reus, Spain; 8 Department of Electronic Engineering, Rovira and Virgili University (URV), Institut d'Investigació Sanitària Pere Virgili (IISPV), Tarragona, Spain; 9 Rheumatology department, “Sant Joan” University Hospital, Reus, Spain; 10 Lipids and Arteriosclerosis Research Unit, Rovira and Virgili University (URV), Reus, Spain; 11 Pere Virgili for Health Research Institute (IISPV), Tarragona, Spain

**Keywords:** lipidomics, metabolomics, COVID-19, HDL, NMR, pneumonia

## Abstract

This prospective observational study compared the 1H NMR blood lipidomes and metabolomes of 71 patients with community-acquired pneumonia (CAP), 75 patients with COVID-19 pneumonia, and 75 healthy controls (matched by age and sex) to identify potential biomarkers and pathways associated with respiratory infections. Both pneumonia groups had comparable severity indices, including mortality, invasive mechanical ventilation, and intensive care unit admission rates. Patients with COVID-19 pneumonia exhibited more pronounced hypolipidemia, with significantly lower levels of total cholesterol and LDL-c compared to patients with CAP. Atherogenic lipoprotein subclasses (VLDL-cholesterol, IDL-cholesterol, IDL-triglyceride, and LDL-triglyceride/LDL-cholesterol) were significantly increased in severe cases of both pneumonia types, while lower HDL-c and small, dense HDL particles were associated with more severe illness. Both infected groups showed decreased esterified cholesterol and increased triglycerides, along with reduced phosphatidylcholine, lysophosphatidylcholine, PUFA, omega-3 fatty acids, and DHA. Additionally, infected patients had elevated levels of glucose, lactate, 3-hydroxybutyrate, and acetone, which are linked to inflammation, hypoxemia, and sepsis. Increased levels of branched-chain amino acids, alanine, glycine, and creatine, which are involved in energy metabolism and protein catabolism, were also observed. Neurotransmitter synthesis metabolites like histidine and glutamate were higher in infected patients, especially those with COVID-19. Notably, severe infections showed a significant decrease in glutamine, essential for lymphocyte and macrophage energy. The severity of COVID-19 pneumonia was also associated with elevated glycoprotein levels (glycoprotein A, glycoprotein B, and glycoprotein F), indicating an inflammatory state. These findings suggest that metabolomic and lipidomic changes in pneumonia are connected to bioenergetic pathways regulating the immune response.

Pneumonia, a common acute respiratory infection affecting the alveoli and distal airways, is a major global health problem associated with high morbidity and short- and long-term mortality in all age groups. Data from the 2019 Global Burden of Diseases (GBD) study showed that lower respiratory tract infections, including pneumonia and bronchiolitis, affected 489 million people worldwide (1). In December 2019, a novel coronavirus (severe acute respiratory syndrome coronavirus-2, SARS-CoV-2) emerged as the cause of an outbreak of pneumonia (2). On 11 February 11, 2020, the World Health Organization (WHO) officially named the illness caused by this virus coronavirus disease 2019 (COVID-19) (3). The rapid spread of COVID-19 led to the WHO declaring a global pandemic, which became the most serious public health challenge of this century to date.

The disease can be mild or progress to acute respiratory distress syndrome (ARDS) and septic shock. Over-activation of the immune system by SARS-CoV-2 infection leads to an uncontrolled release of proinflammatory factors, often referred to as a cytokine storm, resulting in host organ damage and severe respiratory failure with a high mortality rate (4). The rapid development and use of effective COVID-19 vaccines successfully controlled this pandemic and greatly reduced the risk of severe illness and death associated with COVID-19. However, owing to its ability to evolve rapidly, the SARS-CoV-2 virus may never be eradicated (5, 6).

Community-acquired pneumonia (CAP) is a potentially serious condition that often requires admission to intensive care. Severe CAP is associated with significant morbidity and mortality, particularly in older adults or those with comorbidities (7, 8). However, severe CAP can also occur in previously healthy young people and is associated with poor outcomes. Approximately 20% of patients with CAP require hospitalization. Typically, 5%–40%. of patients admitted to hospital with a diagnosis of CAP are admitted to intensive care. Several clinical scoring systems are available to assist clinicians in early severity assessment and identification of CAP patients requiring ICU admission, but accurate prognostic biomarkers are still lacking (9, 10, 11).

Severe CAP, like COVID-19, is a complex disease. The causative pathogen generates an overwhelming inflammatory response (12, 13). Recent studies have suggested an association between disease severity and low lipid levels, although the relationship between hyperinflammatory status, immune response, and hypolipidemia has not been clearly investigated (14, 15). The acute-phase response (APR) is a complex host response induced by a variety of mechanisms, including infection, trauma, burns, ischemia, and malignant growth. The APR is accompanied by changes in plasma proteins, including C-reactive protein (CRP), serum amyloid A (SAA), and several cytokines that induce an inflammatory state (16). Metabolic changes consistent with hypertriglyceridemia are typically associated with the APR, as well as significant changes in the levels of other lipids, lipoprotein composition, and oxidation states (8, 17, 18, 19, 20). COVID-19 is associated with the development of cardiometabolic disorders, including dyslipidemia, the imbalance of HDL, triglycerides, and low-density lipoprotein (LDL). SARS-Cov-2 infection is also associated with notable changes in the lipid profile, which has been suggested as a possible biomarker to aid in the diagnosis and management of COVID-19 (21, 22, 23, 24, 25). A better understanding of lipid metabolism in host–pathogen interactions will provide valuable insights into viral and bacterial pathogenesis and facilitate the development of novel prognostic tools and therapeutic targets (26, 27, 28).

In a retrospective and prospective study carried out before the COVID-19 pandemic, we observed that low levels of HDL cholesterol (HDL-c) on admission in patients with CAP were predictive of clinical outcomes (20). Herein, we present the results of a prospective study comparing the 1H NMR blood lipidomes and metabolomes for patients admitted with a diagnosis of CAP, patients with COVID-19 pneumonia, and a non-infected control group in order to identify new pathways and biomarkers associated with the pathogenesis of respiratory infections.

## Material and Methods

### Study population

This prospective cohort study included patients hospitalized for community-acquired pneumonia (CAP) and COVID-19 pneumonia at Sant Joan University Hospital (Reus, Spain). CAP was defined as an acute illness with two or more of the following: fever, chills, cough, sputum production, pleuritic pain, signs of pulmonary consolidation, and a chest radiograph consistent with the diagnosis. Patients with CAP admitted between January 1, 2017, and December 30, 2019, and patients with COVID-19 pneumonia during the first pandemic wave (March 15 to June 30, 2020) were enrolled in the study (29).

All COVID-19 pneumonia patients had a positive test of SARS-CoV-2 infection by reverse transcription-polymerase chain reaction (RT-PCR). The tests were performed using the VIASURE SARS-CoV-2 Real-Time PCR Detection Kit (CerTest Biotec), and the analyses were performed on a 7,500 Fast RT-PCR System (Applied Biosystems, Foster City, CA, USA). Inclusion criteria were patients over 18 years of age with a diagnosis of CAP on admission to the Internal Medicine Department at Sant Joan University Hospital who agreed to participate in the study and signed the informed consent. We excluded pregnant women, patients with immunodepression due to HIV infection or transplantation, and patients with active oncologic disease or other immune disorders. Patients not fulfilling the inclusion criteria or with nosocomial pneumonia were not included. The collected data included: demographics; comorbidities; clinical findings; vital signs; blood analyses; chest radiographs; microbiology tests; PSI and CURB-65 on admission; and clinical evolution during admission and treatments. The criteria for severity were the need to be in the ICU, mechanical ventilation, or death. A serum sample was collected no later than 24–48 h after admission to the hospital.

As the control group, 75 subjects with no prior diagnosis of infectious, autoimmune, or neoplasic diseases were recruited from the Vascular Medicine and Metabolism Unit of our hospital, where they were seeking care for lipid metabolism disturbances. All the participants gave written informed consent. To ensure comparability, the COVID-19 pneumonia patients, CAP patients, and a control group of non-infected patients were age- and sex-matched. The study was approved by the Clinical Research Ethics Committee of our hospital with reference CEIm: 081/2020 for the infected patients and CEIm: 222/2020 for the control group. The investigation was executed in accordance with our institution’s guidelines and the Declaration of Helsinki.

### Lipoprotein analysis by ^1^H NMR spectroscopy (extended lipoprotein profile)

Frozen samples were shipped on dry ice to Biosfer Teslab for ^1^H NMR analysis. Prior to ^1^H NMR analysis, 200 μl of serum was diluted with 50 μl of deuterated water (D_2_O) and 300 μl of 50 mM phosphate-buffered saline (PBS) at pH 7.4 in a 5-mm glass NMR tube. ^1^H NMR spectra were recorded at 306 K on a Bruker Avance III spectrometer (Bruker BioSciences Española S.A., Rivas Vaciamadrid) operating at a proton frequency of 600.20 MHz (14.1 T). The lipoprotein profile was analyzed using the NMR-based Liposcale® assay. Lipid concentrations (i.e., triglycerides and cholesterol), size and particle number of the four major lipoprotein classes (intermediate-density lipoprotein [IDL], very-low-density lipoprotein [VLDL], LDL, and HDL), and particle number of nine subclasses (large, intermediate, and small VLDL, LDL, and HDL) were determined as previously reported (24). Lipid volumes were determined by using common conversion factors to convert concentration units to volume units. The particle concentration of each subclass was calculated by dividing the lipid volume by the particle volume of a given class. Finally, weighted average VLDL, LDL, and HDL particle sizes were calculated from different subclass concentrations by summing the known diameter of each subclass multiplied by its relative percentage of the subclass particle number (30).

### Analysis of lipid families

After ^1^H NMR metabolomic characterization, the diluted serum samples were lyophilized and then diluted with 100 μl of 50 mM PBS at pH 7.4 before lipid extraction using the butanol and methanol extraction (BUME) method with slight modifications. BUME was optimized for batch extractions with di-isopropyl ether (DIPE) replacing heptane as the organic solvent. This procedure was carried out using a BRAVO liquid handling robot, which can extract 96 samples at a time. The upper lipophilic phase was dried by heating under a vacuum in a Speedvac until the organic solvents had completely evaporated and then stored at −80°C until NMR analysis. Lipid extracts were reconstituted in a solution of CDCl_3_:CD_3_OD:D_2_O (16:7:1, v/v/v) containing tetramethylsilane (TMS) at 1.18 mM as a chemical shift reference and transferred to 5-mm NMR glass tubes. ^1^H NMR spectra were recorded at 286 K using an Avance III 600 MHz Bruker spectrometer at a proton frequency of 600.20 MHz. A 90° pulse with a water presaturation sequence (ZGPR) was used. Quantification of lipid signals in the ^1^H NMR spectra was performed using LipSpin (31), a Matlab-based program that was developed in-house. Resonance assignments were made based on literature values (32).

### Glycoprotein profiling using ^1^H NMR spectroscopy

Glycoprotein profiling was achieved by analyzing the region of the ^1^H NMR spectrum where the glycoproteins resonate (2.15–1.90 ppm) using several analytical functions according to a previously published procedure. The height/width (H/W) ratios of the peaks for GlycA and GlycB were also reported as a parameter related to the aggregation state of the sugar–protein linkages. Height was calculated as the distance from the baseline to the maximum of the corresponding NMR peaks, and width corresponds to the peak width at half height (33).

### Analysis of low-molecular-weight metabolites

Low-molecular-weight metabolites (LMWMs) were identified and quantified in the 1D Carr–Purcell–Meiboom–Gill (CPMG) ^1^H NMR spectra using the line shape fitting approach and peak deconvolution. Several databases (Bioref AMIX database [Bruker], Chenomx, and HMDB (34)), and literature comparison were used for 1D resonance assignment and metabolite identification (35).

### Statistical analysis

Quantitative results are expressed as the median and 25–75 interquartile range, while qualitative or dichotomous variables are presented as percentages. To compare proportions and study relationships between qualitative variables, we used the Chi-square test (χ2) and Fisher’s exact test, depending on the size and characteristics of the variables. To analyze the association between two quantitative variables, we employed the Kruskall–Wallis test. Additionally, Spearman’s correlation coefficients were calculated to assess correlations between parameters.

Random forest analysis was used to identify important variables that distinguish between groups. The top 10 most important variables were determined based on their variable importance scores. To ensure the model’s generalizability and prevent overfitting, 10-fold cross-validation was performed. The data was randomly divided into training (75%) and test (25%) sets. During the model construction process, we conducted a 10-fold cross-validation with 100 replicates on the training data and evaluated the model’s performance on the hold-out test data. We constructed a linear fitting model and evaluated its ability to discriminate between groups using the area under the curve (AUC) of the receiver operating characteristics (ROC) curve, along with a 95% confidence interval. Additionally, 25% of the other individuals were used to externally validate the model, as they were not included in the training dataset's input, thus serving as an independent cohort.

The statistical analysis was conducted using R statistical software version 4.1.1.

## Results

### General characteristics of the study population

A total of 221 patients were eligible to be included in this study. The study population was divided into three groups: healthy controls (n = 75), patients diagnosed with non-COVID pneumonia (n = 71), and patients diagnosed with COVID-19 pneumonia (n = 75) matched by age and sex. The characteristics are shown in Table 1.

**Table 1:** General characteristics of the healthy control, CAP and COVID-19 pneumonia groups

Variable	Control	CAP	COVID-19 Pneumonia	p.Overall	p.Control versus CAP	p.Control versus COVID-19	p.CAP versus COVID-19
	N = 75	N = 71	N = 75				
Age (y)	64.2 (12.4)	66.0 (20.9)	64.4 (12.8)	0.751	0.761	0.993	0.821
Sex:				0.836	1.000	1.000	1.000
Female	31 (41.3%)	26 (36.6%)	30 (40.0%)				
Male	44 (58.7%)	45 (63.4%)	45 (60.0%)				
Diabetes mellitus	13 (17.3%)	13 (18.3%)	19 (25.3%)	0.417	1.000	0.614	0.614
Hypertension	18 (27.7%)	36 (51.4%)	44 (58.7%)	0.001	0.013	0.001	0.479
Dyslipidemia	7 (16.3%)	14 (20.9%)	9 (22.0%)	0.777	1.000	1.000	1.000
Obesity	11 (14.7%)	23 (32.4%)	17 (22.7%)	0.001	0.002	0.295	0.032
Current smokers	11 (25.6%)	24 (35.8%)	4 (5.33%)	<0.001	0.061	<0.001	<0.001
Hepatic disease	0 (0.00%)	0 (0.00%)	1 (1.33%)	1.000	.	.	1.000
Cardiovascular disease	0 (0.0.%)	11 (15.5%)	38 (50.7%)	<0.001	.	.	<0.001
Chronic obstructive pulmonary disease	0 (0.0.%)	20 (28.2%)	13 (17.3%)	0.172	.	.	0.172
Neurological disease	0 (0.0.%)	5 (7.04%)	12 (16.0%)	0.153	.	.	0.153
Chronic kidney disease	0 (0.0.%)	10 (14.1%)	10 (13.3%)	1.000	.	.	1.000
Immunodeficiency	0 (0.0.0%)	3 (4.23%)	1 (1.33%)	0.356	.	.	0.356
Charlson Comorbidity Index:				0.085	.	.	0.085
0	0 (0.0.%)	26 (36.6%)	35 (46.7%)				
1	0 (0.0.%)	11 (15.5%)	16 (21.3%)				
2	0 (0.0.%)	16 (22.5%)	19 (25.3%)				
3	0 (0.0.%)	11 (15.5%)	4 (5.33%)				
4	0 (0.0%)	5 (7.04%)	1 (1.33%)				
5	0 (0.0%)	1 (1.41%)	0 (0.00%)				
7	0 (0.0%)	1 (1.41%)	0 (0.00%)				
Neoplasm	0 (0.0%)	8 (11.3%)	11 (14.7%)	0.716	.	.	0.716
Radiographic extent:				<0.001	.	.	<0.001
No condensation	0 (0.0%)	5 (7.04%)	17 (30.4%)				
Lobar	0 (0.0%)	35 (49.3%)	6 (10.7%)				
Bilobar	0 (0.0%)	16 (22.5%)	2 (3.57%)				
Bilateral	0 (0.0%)	15 (21.1%)	31 (55.4%)				
Clinical severity							0.734
Moderate	.	58 (70.4%)	50 (70.4%)				
Severe	.	17 (22.6%)	17 (23.9%)				
ICU	0 (0.0%)	13 (19.4%)	12 (15.4%)	1.000	.	.	1.000
Death	0 (0.0%)	6 (8.82%)	12 (15.4%)	0.608	.	.	0.608
IMV	0 (0.0%)	8 (11.8%)	2 (15.4%)	0.659	.	.	0.659

ICU, intensive care unit; IMV, intensive mechanical ventilation.

There were no differences between the three groups in terms of dyslipidemia or diabetes. Regarding hypertension, the patients infected with COVID-19 (58.7%) and CAP (51.4%) presented significantly higher rates of hypertension compared to the control group (22.7%; *P* < 0.001). Obesity was more prevalent in the patients with CAP (43.4%) than in the control (14.7%; *P* < 0.001) and COVID-19 groups (22.7%; *P* < 0.002). These results show that in our age and sex-matched cohort of patients, hypertension is a risk factor for both COVID-19 infection and CAP, where obesity is more prevalent than in COVID-19 patients. In our study, we also found significantly higher rates of cardiovascular disease (50.7%) in COVID-19 patients than in CAP patients (15.5%; *P* < 0.001).

Both groups of pneumonia patients were comparable regarding severity indexes with no significant differences in the rates of mortality, invasive mechanical ventilation (IMV), and admission to the ICU. As expected, the radiologic patterns differed between the two pneumonia groups, with a greater proportion of patients with bilateral pneumonia (55.4%) in the COVID-19 group compared to the CAP group (21.1%); the CAP group also had a higher incidence of lobar pneumonia (49.3%).

In summary, we found that hypertension is a risk factor for both groups of age- and sex-matched pneumonia patients, but cardiovascular disease was more prevalent in the COVID-19 patients while obesity was more common in the CAP patients.

### Lipoprotein profile

The lipoprotein profiles were analyzed in detail using ^1^H NMR. Firstly, it is noteworthy that, compared to the control group, both the COVID-19 pneumonia and CAP patients showed significantly lower levels of total cholesterol (TC), LDL-cholesterol (LDL-c), and HDL cholesterol (HDL-c), and significantly higher levels of total triglycerides (Tg), IDL-cholesterol (IDL-c), and VLDL-cholesterol (VLDL-c), a pattern that is known as “atherogenic dyslipidemia” (Table 2).

**Table 2:** Differences in the advanced lipid profiles for the three groups (healthy control, CAP and COVID-19 pneumonia) analyzed by 1H-NMR

Lipoprotein Subclass	Control N = 75	CAP N = 75	COVID-19 Pneumonia N = 71	*P*-value All Goups	*P*- value CAP versus COVID-19
Total cholesterol (mg/dl)	226 [208;247]	184 [170;204]	173 [155;194]	<0.001	0.004
LDL-cholesterol (mg/dl)	136 [121;152]	99.7 [89.5;120]	94.5 [78.7;114]	<0.001	0.014
HDL cholesterol (mg/dl)	62.3 [55.7;71.4]	43.0 [32.1;51.4]	37.6 [31.4;43.7]	<0.001	NS
Total triglycerides (mg/dl)	87.6 [70.8;113]	134 [108;173]	142 [113;173]	<0.001	NS
VLDL-cholesterol (mg/dl)	12.1 [8.42;18.3]	20.0 [13.6;28.2]	21.4 [16.2;26.1]	<0.001	NS
IDL-cholesterol (mg/dl)	9.78 [7.54;13.1]	18.4 [13.6;24.0]	17.6 [13.6;20.4]	<0.001	NS
VLDL-triglycerides (mg/dl)	49.1 [37.7;69.8]	70.9 [55.9;105]	84.4 [67.5;107]	<0.001	0.036
IDL-triglycerides (mg/dl)	10.7 [8.84;13.1]	17.1 [14.0;21.7]	16.9 [14.5;19.6]	<0.001	NS
LDL-triglycerides (mg/dl)	14.4 [12.1;17.1]	21.3 [17.1;25.9]	19.9 [16.5;24.5]	<0.001	NS
HDL-triglycerides (mg/dl)	12.6 [10.9;15.1]	19.0 [15.6;21.0]	17.9 [16.1;21.0]	<0.001	NS
VLDL-P (nM)	36.9 [27.3;51.6]	53.2 [43.0;78.8]	61.9 [49.4;81.3]	<0.001	NS
Large VLDL-P (nM)	0.98 [0.71;1.28]	1.42 [1.13;1.82]	1.66 [1.31;1.97]	<0.001	0.007
Medium VLDL-P (nM)	4.19 [3.30;5.73]	5.23 [3.24;7.09]	5.56 [4.58;6.98]	0.004	NS
Small VLDL-P (nM)	32.5 [22.9;45.4]	47.3 [37.7;71.5]	54.3 [42.2;71.8]	<0.001	NS
LDL-P (nM)	1,309 [1,166;1,488]	1,089 [963;1,279]	1,023 [839;1,193]	<0.001	0.011
Large LDL-P (nM)	219 [195;235]	171 [154;200]	162 [143;184]	<0.001	0.013
Medium LDL-P (nM)	408 [335;471]	331 [259;432]	314 [236;386]	<0.001	NS
Small LDL-P (nM)	684 [611;806]	582 [529;672]	545 [480;619]	<0.001	0.007
HDL-P (μM)	30.2 [27.6;33.7]	20.4 [16.6;25.7]	19.7 [16.6;22.7]	<0.001	NS
Large HDL-P (μM)	0.31 [0.29;0.34]	0.31 [0.29;0.35]	0.30 [0.27;0.34]	NS	NS
Medium HDL-P (μM)	11.1 [9.88;12.9]	11.1 [10.3;12.7]	10.3 [9.73;11.2]	0.006	0.008
Small HDL-P (μM)	18.7 [16.9;21.6]	8.69 [5.68;13.1]	9.08 [5.79;11.6]	<0.001	NS
VLDL diameter (nm)	42.2 [42.1;42.4]	42.0 [41.8;42.2]	42.1 [41.9;42.2]	<0.001	NS
LDL diameter (nm)	21.2 [21.0;21.3]	21.1 [20.9;21.3]	21.1 [21.0;21.2]	NS	NS
HDL diameter (nm)	8.29 [8.25;8.33]	8.54 [8.45;8.69]	8.49 [8.42;8.65]	<0.001	NS
Non-HDL-P (nM)	1,337 [1,186;1,515]	1,159 [1,017;1,335]	1,086 [943;1,255]	<0.001	0.013
Total P/HDL-P	1.04 [1.03;1.05]	1.06 [1.05;1.09]	1.05 [1.05;1.07]	<0.001	0.036
LDL-P/HDL-P	4.15 [3.73;4.63]	3.60 [3.11;4.16]	3.65 [2.99;4.04]	<0.001	NS
VLDL-TG/VLDL-C	4.13 [3.79;4.92]	4.09 [3.64;4.48]	4.06 [3.83;4.46]	NS	NS
IDL-TG/IDL-C	1.09 [1.00;1.16]	0.96 [0.88;1.06]	0.98 [0.92;1.05]	<0.001	NS
LDL-TG/LDL-C	0.10 [0.09;0.12]	0.20 [0.16;0.26]	0.21 [0.17;0.26]	<0.001	NS
HDL-TG/HDL-C	0.18 [0.15;0.25]	0.43 [0.33;0.61]	0.48 [0.39;0.65]	<0.001	NS

The COVID-19 pneumonia patients exhibited more pronounced hypolipidemia than the CAP patients. COVID-19 pneumonia patients showed lower levels of TC, [173 (155–194) mg/dl versus 184 (170–204) mg/dl, *P* = 0.004]; and LDL-c [94.5 (78.7–114) mg/dl versus 99 (89–120) mg/dl, *P* = 0.014].

**Fig. 1:**
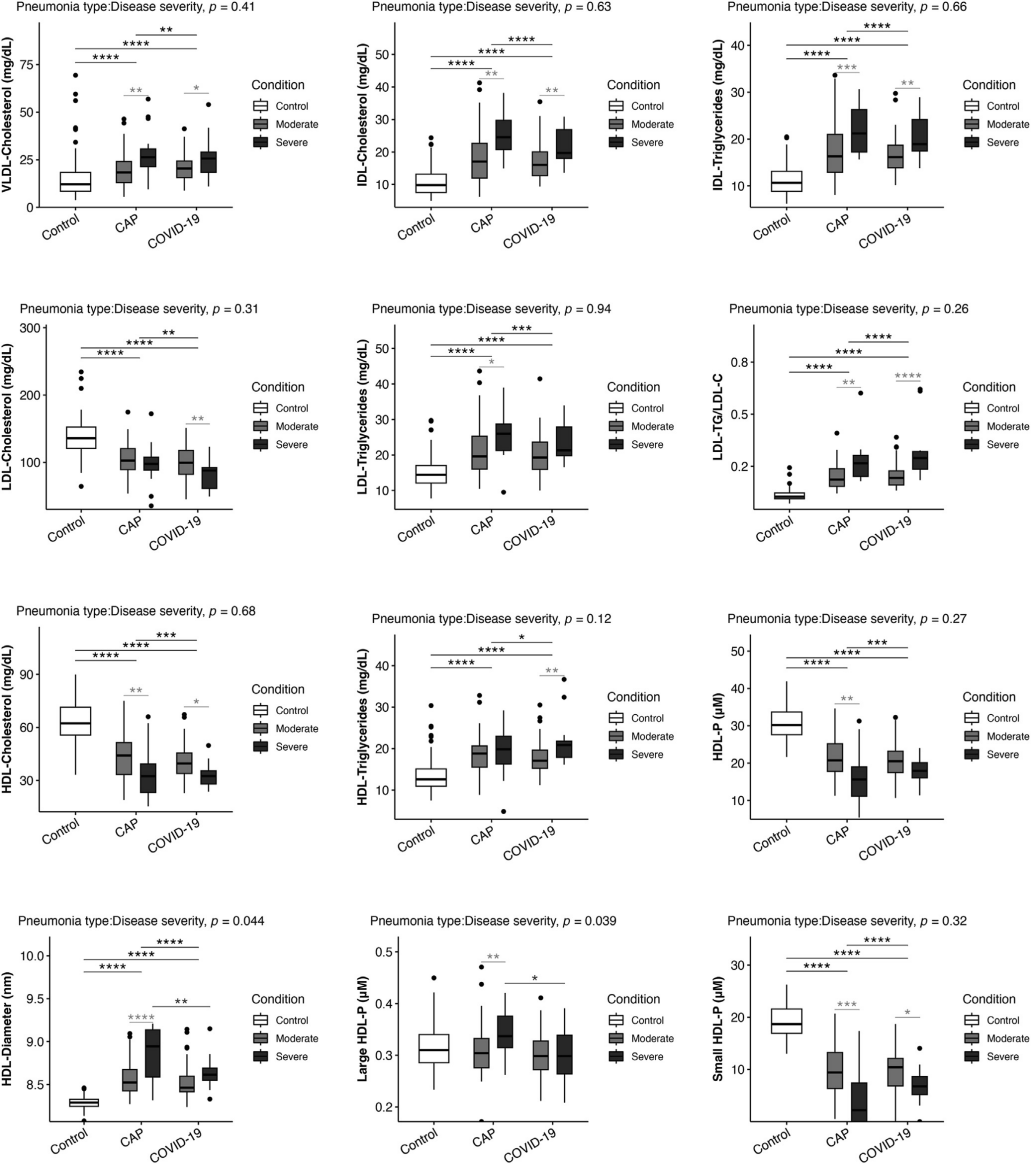
Lipoprotein subclasses for the control, COVID pneumonia and CAP based on clinical severity. The data presented in the figures represent medians with quartiles. Statistical analysis was performed using T-tests to compare disease severity (light grey) and pneumonia etiology (dark grey), ANOVA to assess group differences (black), and two-way ANOVA to analyze the interaction between pneumonia type and disease severity. Significance levels are denoted as follows: ∗*P* < 0.05, ∗∗*P* < 0.01, ∗∗∗*P* < 0.001.

The lipoprotein subclasses were analyzed separately for both types of pneumonia and according to clinical severity (Fig. 1). The levels of proatherogenic particles VLDL-c, IDL-c, IDL-tg, and LDL-tg/LDL-c were higher in the COVID-19 pneumonia group than in the CAP group, but they were also significantly higher for patients with a more severe presentation for both types of pneumonia.

Interestingly, the decreased LDL-c levels are reflected by the significantly decreased numbers of total LDL particles and large, medium, and small LDL particles in both pneumonia groups compared to the control group. There was an increase in the total number of LDL particles enriched by triglycerides (LDL-tg) (Table 2). The LDL-tg/LDL-c ratios for both the pneumonia groups were higher than that of the control group (*P* < 0.001), and this ratio was also higher in the COVID-19 pneumonia group than the CAP group, and in the more severe patients in both groups (Fig. 1).

The HDL-c levels were lower in the COVID-19 pneumonia group than in the CAP group [37.6 (31.4–43.7) mg/dl versus 43 (31.1–51.4) mg/dl] and it is noteworthy that in both groups the levels were much lower than for the control group 62.3 (55.7–71.4) mg/dl; (*P* < 0.001) (Table 2). The significantly lower HDL-c level in both groups of infected patients is also reflected by the analyses of the lipoprotein subpopulations, which revealed a decrease in the total number of HDL particles, mainly small HDL particles, not only in both pneumonia groups compared to the control group, but also, and more pronounced in severe patients in both groups of infected patients (Fig. 1).

In summary, infected patients showed hypolipidemia with increased levels of triglyceride-carrying particles, reflecting increased lipid catabolism in response to infection that is more pronounced in COVID-19 patients.

When we analyzed the lipid subpopulations in terms of severity in CAP and COVID-19 pneumonia patients, we again found that the severity (admission to ICU and/or IVM) was associated with significantly increased levels of atherogenic lipoprotein subclasses, VLDL-c, IDL-c, IDL-tg, and LDL-tg/LDL-c. Conversely, lower levels of HDL-c and the small, dense HDL particles were associated with more severe illness in both pneumonia groups.

### Lipid families

**Fig. 2:**
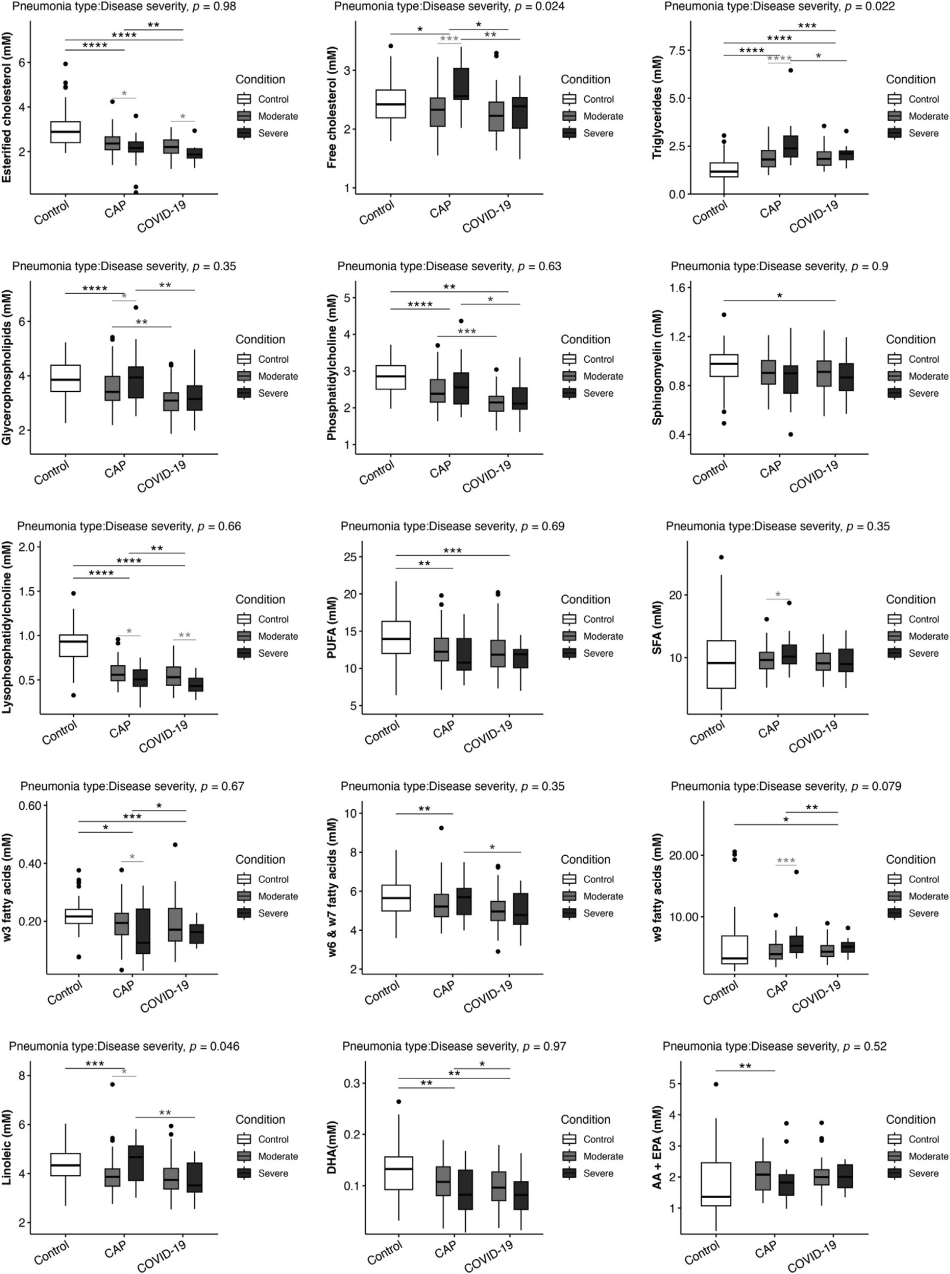
Lipid components differentiated by pneumonia severity. The data presented in the figures represent medians with quartiles. Statistical analysis was performed using T-tests to compare disease severity (light grey) and pneumonia etiology (dark grey), ANOVA to assess group differences (black), and two-way ANOVA to analyze the interaction between pneumonia type and disease severity. Significance levels are denoted as follows: ∗*P* < 0.05, ∗∗*P* < 0.01, ∗∗∗*P* < 0.001.

We compared the lipid profiles for the healthy control group and the two groups of pneumonia patients separated by clinical severity (patients in the ICU and/or IVM). The results are shown in Fig. 2. As expected, both groups of infected patients showed significant differences regarding the lipid components, in agreement with the results of the lipoprotein analyses. We observed the same decreased esterified cholesterol and increased triglyceride levels that we observed with the lipoprotein subclasses in the two groups of infected patients compared to the healthy group.

In the same way, we observed significantly lower levels of phosphatidylcholine, lysophosphatidylcholine, polyunsaturated fatty acids (PUFA), w3 fatty acids, and DHA (docohexaenoic acid) in both groups of infected patients compared to the healthy group.

The levels of esterified and free cholesterol, triglycerides, glycerophospholipids, phosphatidylcholine, lysophosphatidylcholine, w3, w6, and w7, and DHA were significantly lower in the COVID-19 pneumonia patients than in the CAP patients.

Regarding clinical severity in the more patients with severe CAP, we observed significantly lower levels of esterified cholesterol, lysophosphatidylcholine, and w3 and significantly higher levels of free cholesterol, triglycerides, glycerophospholipids, saturated fatty acids (SFA), and w9 compared to those in the less severe and control groups (Fig. 2).

With respect to clinical severity in the COVID-19 pneumonia patients we only found differences in the levels of esterified cholesterol and lysophosphatidylcholine in the same way as the severe CAP patients.

### Low-molecular-weight metabolite profile

**Fig. 3:**
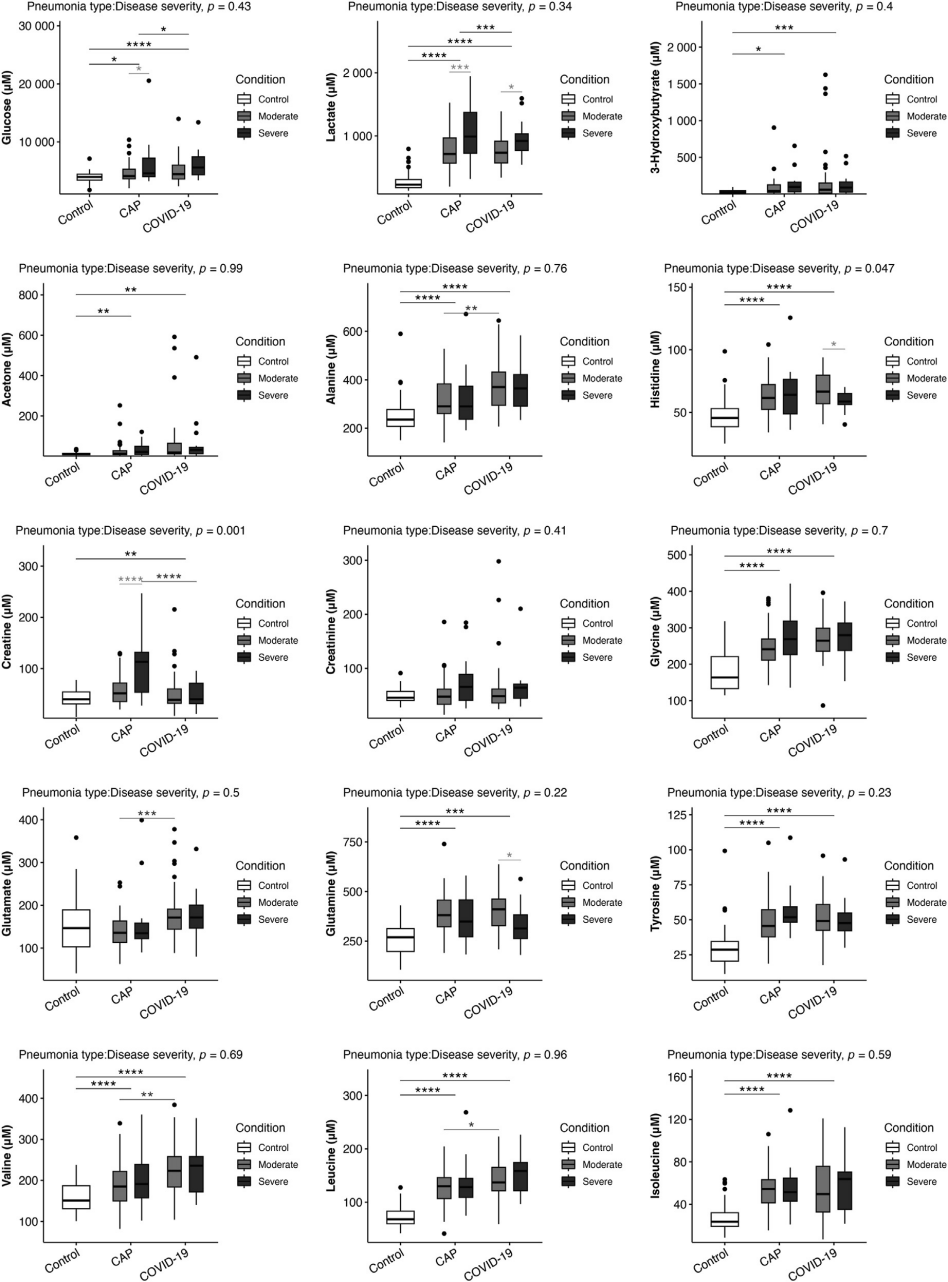
Low-molecular-weight metabolite profile. The data presented in the figures represent medians with quartiles. Statistical analysis was performed using T-tests to compare disease severity (light grey), pneumonia etiology (dark grey), ANOVA to assess group differences (black), and two-way ANOVA to analyze the interaction between pneumonia type and disease severity. Significance levels are denoted as follows: ∗*P* < 0.05, ∗∗*P* < 0.01, ∗∗∗*P* < 0.001.

The metabolomic analysis adds information to the lipid profile and allows us to explore the interactions between lipids, low-molecular-weight metabolites, and inflammatory status. The results are presented in Fig. 3.

The LMWM profile for the healthy control group was significantly different to the profiles for the two groups of infected patients, except for the creatinine levels, which did not differ between groups.

The infected groups exhibited significantly elevated levels of glucose, lactate, 3-hydroxybutyrate, and acetone, which are all associated with inflammatory status, hypoxemia, and sepsis, and increased levels of the branched-chain amino acids (BCAAs) valine, leucine, and isoleucine, which play a role in energy metabolism and protein synthesis.

Another interesting result is the increased levels of alanine in both of the infected groups. Alanine is involved in energy production and the transport of amino acids from muscle tissue to the liver for conversion to glucose. We also found that glycine, a non-essential amino acid that is also involved in protein synthesis, was increased in both infected groups.

Histidine, which is involved in the synthesis of histamine, is a neurotransmitter and an inflammatory mediator, as is tyrosine, which is the precursor to neurotransmitters such as dopamine, norepinephrine, and thyroid hormones. Both histidine and tyrosine were significantly elevated in both groups of pneumonia patients.

Patients with severe CAP had significantly higher levels of glucose, lactate, and creatine than those with moderate CAP. Creatine is not an amino acid, but it is a metabolite that is primarily stored in muscle tissue and plays a role in energy production during short bursts of high-intensity activity.

In the patients with severe COVID-19 pneumonia, we also found higher levels of lactate compared to those in moderate patients, as with the CAP group, but there was a significant decrease in the levels of histidine and glutamine. Glutamine is the most abundant free non-essential amino acid in the bloodstream. Glutamine plays several important roles, such as in protein synthesis and as an energy source in the liver through gluconeogenesis, especially during times of fasting or intense physical activity. It is essential as an energy source for lymphocytes and macrophages and for the integrity of the intestinal mucosa, antioxidant properties, muscle recovery, and brain function.

Another interesting result was noted for the metabolite glutamate (an excitatory neurotransmitter in the central nervous system), which was significantly higher in the COVID-19 pneumonia group than in both the control group and the non-COVID pneumonia group.

### Inflammatory status measured by plasma glycoproteins

**Fig. 4:**
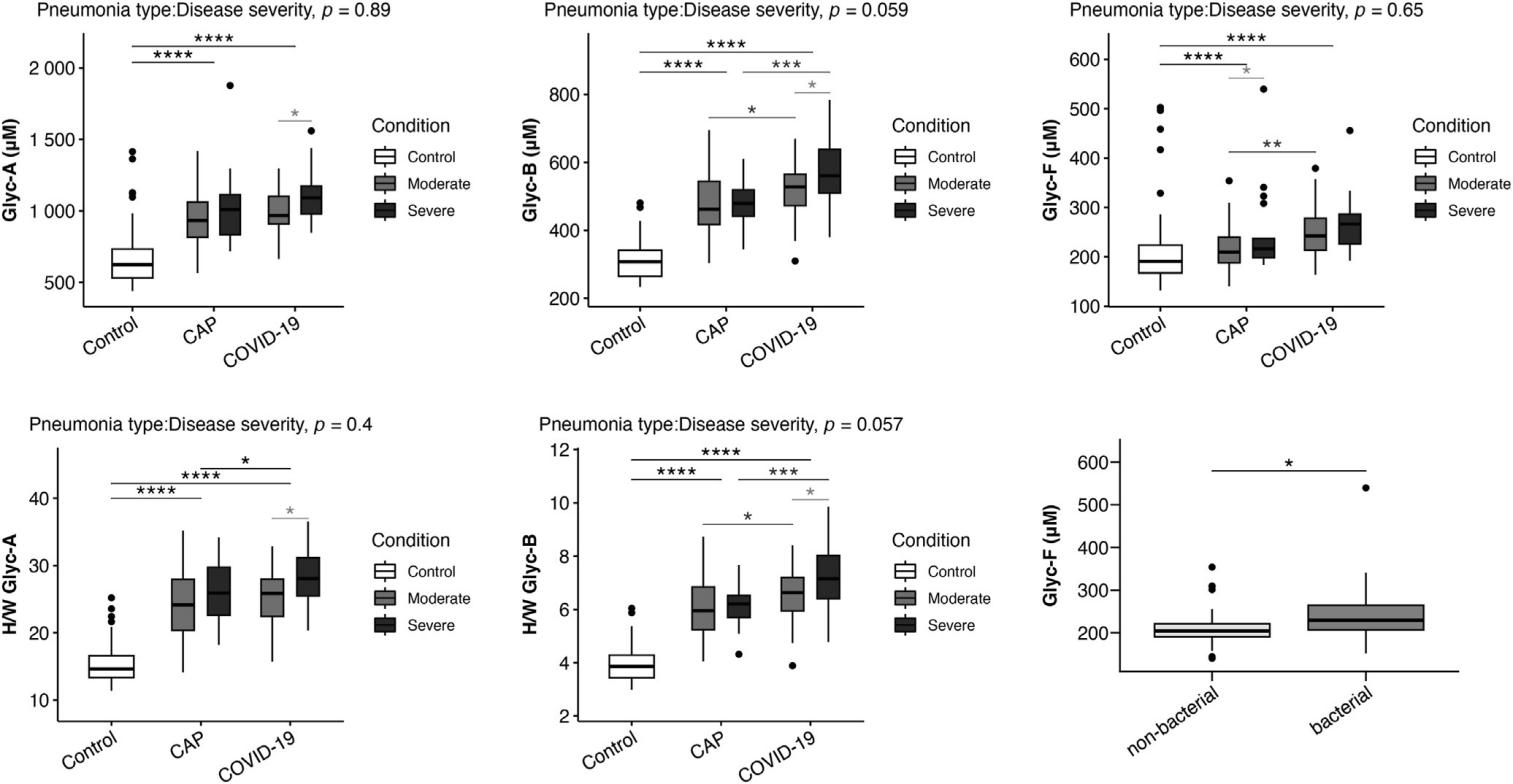
Glycoproteins. The data presented in the figures represent medians with quartiles. Statistical analysis was performed using T-tests to compare disease severity (light grey), pneumonia etiology (dark grey), ANOVA to assess group differences (black), and two-way ANOVA to analyze the interaction between pneumonia type and disease severity. Significance levels are denoted as follows: ∗*P* < 0.05, ∗∗*P* < 0.01, ∗∗∗*P* < 0.001.

Patients with both types of pneumonia had elevated levels of glycoproteins, as determined by ^1^H NMR, compared to the healthy control group. This is indicative of an inflammatory state, with COVID-19 pneumonia patients exhibiting the highest glycoprotein levels (Fig. 4).

The degree of severity was also associated with higher glycoprotein levels in patients with COVID-19 pneumonia (GlycA, GlycB, and GlycF). While glycoprotein levels did not show a statistically significant increase based on severity in CAP patients, the glycoprotein GlycF was significantly elevated in CAP patients with bacterial etiology.

**Fig. 5:**
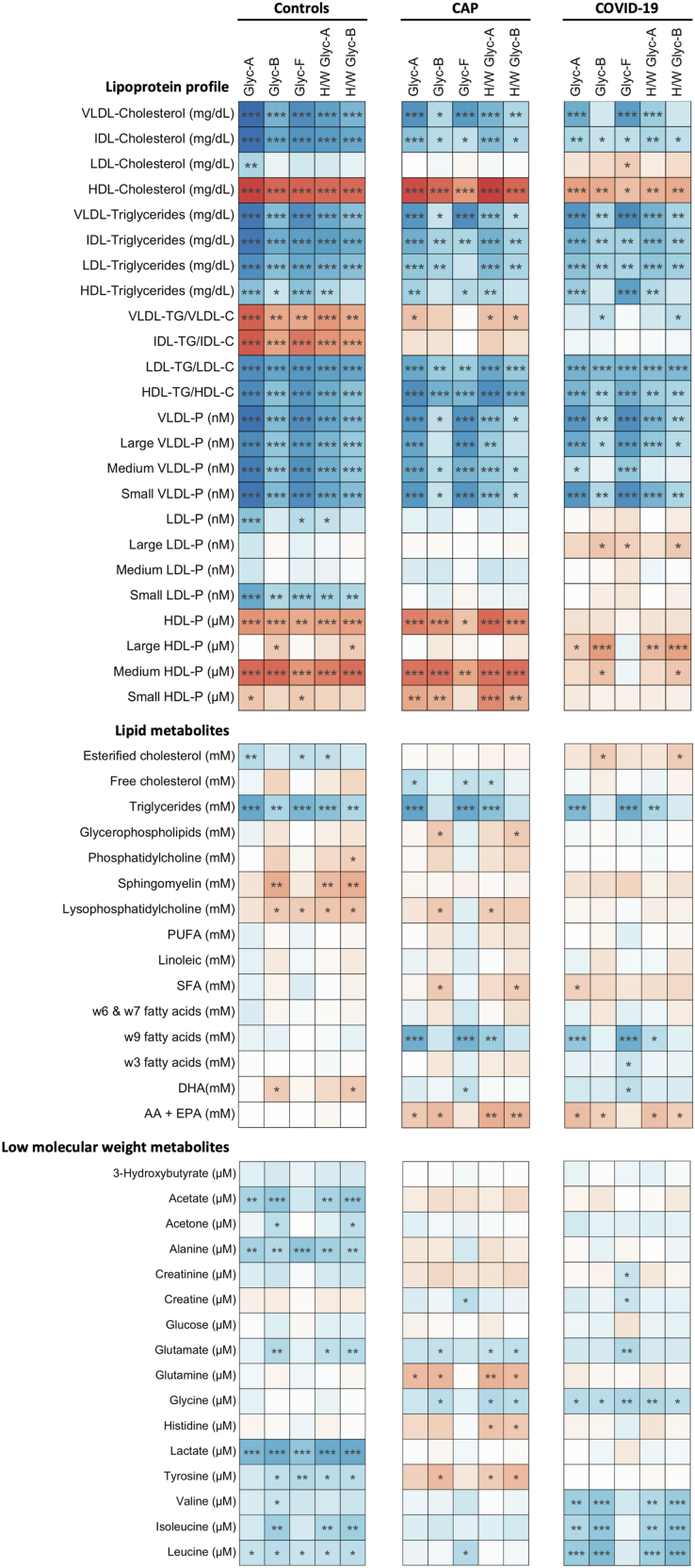
Bivariate correlations between glycoproteins and lipoprotein profiles, lipid metabolites, and LMWM.

The interactions between the glycosylation peaks, the lipoprotein profiles, the lipid families, and the LMWM are shown in Fig. 5 and supplemental Fig. S2. Briefly, glycoprotein levels were positively correlated with proatherogenic lipoproteins and triglycerides, and a negative correlation with the HDL-c and HDL particles. Regarding lipid metabolites, there was a positive correlation between triglycerides and w9 fatty acids, whereas lysophosphatidylcholine, sphingomyelin, and AA + EPA showed an inverse correlation with glycosylation peaks.

Differences in correlations between the LMWM and glycosylation peaks were evident across the groups. In the control group, lactate showed the strongest positive association with glycosylation. Conversely, the CAP group displayed an inverse correlation between glycosylation and the levels of glutamine and tyrosine. In the group of COVID-19 patients, an association was found between glycosylation and the levels of glutamate, glycine, and BCAAs (valine, isoleucine, and leucine), indicating heightened inflammation.

### Multivariate analyses

**Fig. 6:**
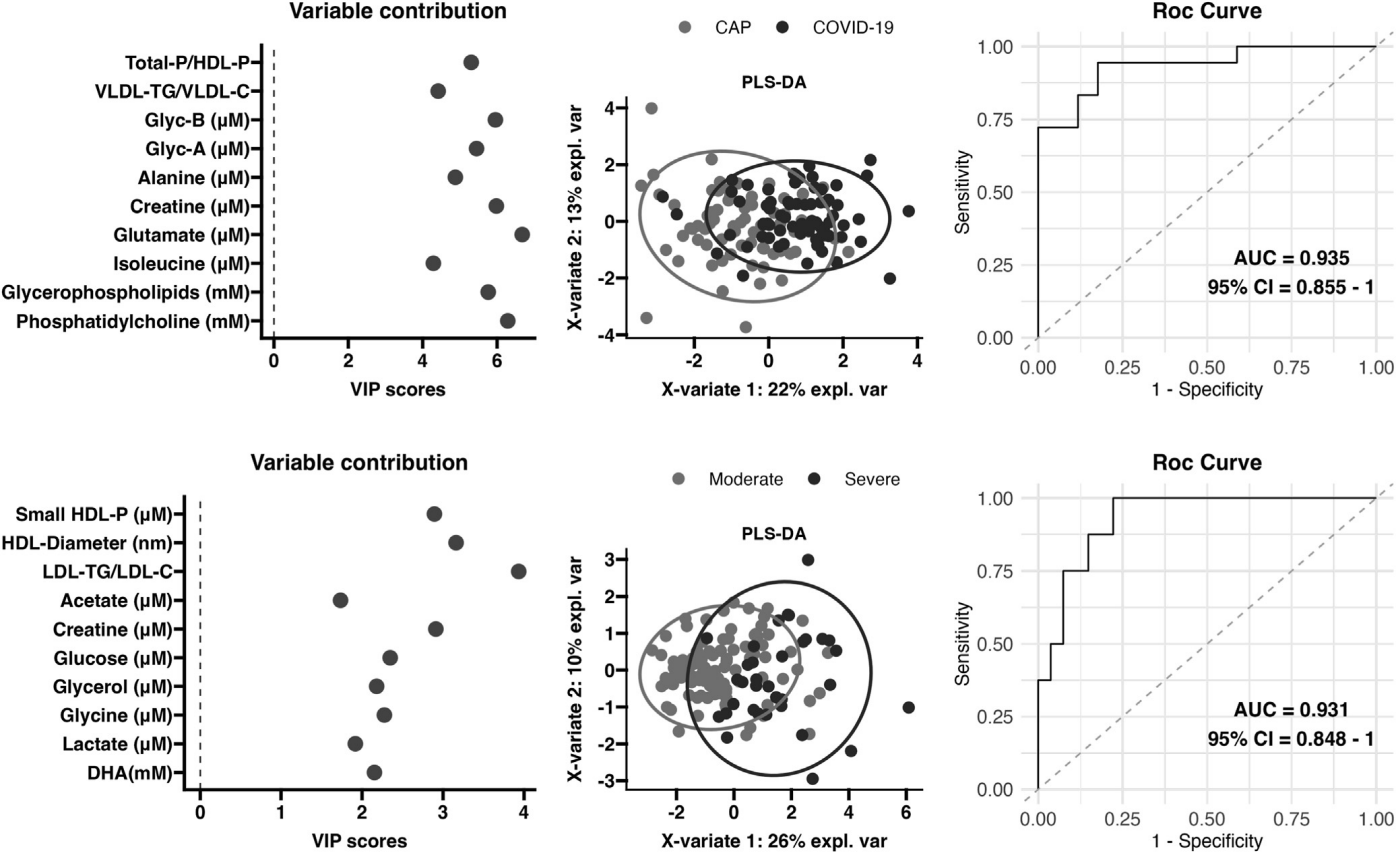
Multivariant analysis. Partial least squares discriminant analysis (PLS-DA) and ROC curve analysis performed: CAP/COVID-19 pneumonia and moderate/severe.

To gain critical insight into the metabolomic profiles associated with the different types and severities of pneumonia, we employed a two-pronged multivariate data analysis approach. We used the metabolomic parameters obtained by ^1^H NMR analysis to distinguish between the two different etiologies of pneumonia, and between moderate and severe categories (Fig. 6A, B and supplemental Fig. S1). Our analytical framework involved the identification of the ten most important variables derived from the random forest classification, specific to either etiological or severity-based patient groupings. We then evaluated the discriminatory potential of these selected variables within their respective patient cohorts. To validate the relevance of this variable panel for accurate classification, we applied partial least squares discriminant analysis (PLS-DA) and performed ROC curve analysis. The resulting model for classifying patients according to their etiological factors includes the following variables: Total P/HDL-P, VLDL-TG/VLDL-C, GlycB, GlycA, alanine, creatine, glutamate, isoleucine, glycerophospholipids, and phosphatidylcholine. This model has an AUC of 0.935, with a 95% confidence interval (CI) of 0.855–1. The model has an accuracy rate of 0.886, coupled with a sensitivity of 0.944 and a specificity of 0.824. The *P*-value, indicating that the accuracy exceeds the no-information rate, is 3.72 × 10^−6^. The out-of-bag (OOB) error for this model is 0.233 (Fig. 6A). The developed model for the classification of patients based on severity includes the following variables: small HDL-P, HDL diameter, LDL-TG/LDL-C, acetate, creatine, glucose, glycerol, glycine, lactate, and DHA. This model has an AUC of 0.931, with a 95% CI ranging from 0.848 to 1. The accuracy of the model is 0.829, accompanied by a sensitivity of 1.00 and a specificity of 0.778, and the *P*-value is 2.81 × 10^−1^. In addition, the OOB error value for this model is 0.232 (Fig. 6B).

## Discussion

We present the results of a lipidomic and metabolomic investigation of the metabolic changes associated with COVID-19 pneumonia, and also the differences and similarities with respect to CAP and a healthy control group. It is of interest to study metabolic changes related to the host response to the infection itself. Comparing the metabolic profiles of COVID-19 pneumonia with community-acquired pneumonia (CAP) and healthy controls can help differentiate between these conditions. This is crucial for accurate diagnosis and understanding of how different pathogens affect the host metabolism differently.

In our cohort of patients cardiovascular disease is more prevalent in patients with COVID-19 pneumonia, but hypertension was found to be a risk factor for both groups of patients with pneumonia, matched for age and sex without differences in severity indices, not just COVID-19 pneumonia patients, as previously reported (2, 3, 29). It has also been reported that hypertensive patients are at higher risk of more severe COVID-19 due to the ACE receptors of the well-known “spike” protein of SARS-CoV-2 (36). However, the incidence of cardiovascular disease seems to be higher in the COVID-19 group of patients, which could indicate a higher risk of COVID-19 pneumonia in hosts with preexisting endothelial vascular damage and a proinflammatory status (29, 37). On the other hand, with the same cardiovascular risk factors, in CAP patients there is a higher prevalence of obesity, which could indicate that these patients are at higher risk of more severe pneumonia due to hypoventilatory dysfunction rather than the endothelial damage itself (9, 11).

Both of the infected patient groups exhibited lipid profile changes similar to the characteristic atherogenic dyslipidemia found in COVID-19 patients. This dyslipidemia is also seen in other infections and inflammatory diseases, such as diabetes or autoimmune diseases, suggesting that the cause is the inflammatory condition (14, 18, 22, 23, 38, 39, 40). Our results confirmed that infected patients had lower levels of lipoproteins and higher levels of triglyceride-carrying particles, reflecting increased lipid catabolism during the acute infection. Although hypolipidemia was observed in both of the infected groups, it is more intense in the COVID-19 pneumonia patients, perhaps owing to the presence of the exaggerated inflammatory reaction that has been observed in COVID-19 patients and that in some extreme cases has been attributed to the cytokine storm. Similar results have also been reported from previous studies involving COVID-19 and other pneumonia and have been associated with worse clinical outcomes, especially for patients with lower levels of HDL-c and HDL particles (21, 25, 38, 41, 42, 43, 44, 45).

Lipids are essential components of cell membranes and lipid rafts, which mediate cell receptor expression and immune cell activation. Lipids play a crucial role throughout the viral cycle, and pathogens are known to exploit lipid signaling and synthesis, that affect the host cell lipidome (26, 27, 46, 47). For this reason, research on the effects of host–pathogen interactions on lipid metabolism is of great interest so we performed an in-depth analysis of lipid metabolites. Our results confirmed that both types of pneumonia are associated with a marked decrease in most of the lipid components compared to the control healthy group. This includes esterified cholesterol, which is primarily transported by apolipoproteins. These changes are in line with the observed decrease in lipoproteins and increase in triglycerides and free cholesterol that we found in both pneumonia groups with respect to the control group and significantly lower in the more severe CAP and COVID-19 pneumonia groups.

Phosphatidylcholine and lysophosphatidylcholine levels were also reduced in both of the pneumonia groups, but the COVID-19 pneumonia patients showed the lowest levels. The patients with the most severe CAP and COVID-19 pneumonia had the lowest levels of lysophosphatidylcholine. Phosphatidylcholine and lysophosphatidylcholine, the main phospholipid components of cell membranes, are involved in the inflammatory response to pathogens. They promote macrophage polarization in response to pathogens, promoting cytokine secretion and a prothrombotic state (18, 26, 43, 48, 49). On the other hand, the inflammatory secretory phospholipase A_2_ (sPLA2) catalyzes the breakdown of these membrane phospholipids to produce free fatty acid (arachidonic acid) and lysophospholipids, which are metabolized by autotaxin to produce inflammatory lipid mediators (46). Increased _S_PLA2 activity has previously been linked with the cytokine storm in COVID-19 (48, 50).

The levels of omega-3 fatty acids and their precursors, such as PUFA, linoleic acid, and DHA, were decreased in both groups of infected patients. PUFAs and DHA have been shown to directly modulate lipid rafts and membrane domain properties. An immunonutrition approach based on this could potentially be useful as a therapeutic strategy with potential implications for signaling networks that rely on the compartmentalization of proteins within and outside of rafts as it interferes with the mechanisms through which the virus exploits cell lipid metabolism and replication (47, 51). Omega-3 fatty acids have been associated with the resolution of inflammation and reduced lung damage in ARDS and pneumonia, and have been used as adjuvant therapy in severe infections in patients admitted to the ICU (52, 53, 54).

The lipidomic and metabolomic analyses performed by ^1^H NMR showed that the inflammatory status can be determined through the presence of plasma glycoproteins belonging to the large family of acute phase proteins (APPs) (55). The glycosylation peaks were increased with acute infection. Research in this area is expanding, and several studies have suggested that the glycation patterns of these proteins can lead to cellular changes in a wide range of diseases, making them diagnostic markers (55, 56). Antibody glycosylation has received considerable attention in SARS-CoV-2 infections and more recently in vaccination (57). Antibody glycosylation, and particularly immunoglobulin G1 fucosylation, influences effector functions and is therefore a key parameter for assessing the efficacy and safety of SARS-CoV-2 immune responses after vaccination.

This study aimed to generate a comprehensive, high-quality, integrated metabolomic and lipidomic dataset for COVID-19 pneumonia and CAP that can be used to identify potential biomarkers for disease diagnosis and prognosis. The results will also contribute to better understanding of the pathogenesis of respiratory infections and could be used to guide the development of personalized treatments. The results from the present study showed that there are similar alterations in the LMWM profiles in both infections, not just in COVID-19, as had been reported before (45, 58, 59, 60, 61).

We developed a model based on alanine, creatine, glutamate, and isoleucine that could distinguish COVID-19 pneumonia patients from CAP patients. Severe and moderate pneumonia could be distinguished using a model based on acetate, creatine, glucose, glycerol, glycine, and lactate. These findings suggest that oxygen homeostasis and the response to hypoxia are altered, mainly mediated by glycolysis and the lactate cycle in mitochondria, but also involving the pathways of protein synthesis (isoleucine) and neuroinflammation (glutamate, glycine).

The challenge, and one of the limitations of this work, is that metabolomic and lipidomic studies are very complicated when it comes to integrating and interpreting such a large amount of data and results. Nevertheless, the results of our study are quite comprehensive if we summarize that the identified metabolites are essential for the maintenance of a condition that has recently been termed “endurance” or “stamina” in sports medicine. Stamina is the physiological and metabolic capacity of an organism to sustain prolonged physical or mental effort and is highly dependent on the efficient utilization of oxygen and energy substrates (such as carbohydrates and lipids) by the body’s cells to produce ATP, the primary energy source for immune cell activation (62). This is concordant with the persistent symptoms and risk of complications experienced by patients with COVID-19 pneumonia and CAP (5, 28, 63).

In conclusion, the metabolomic and lipidomic changes in patients with COVID-19 pneumonia or CAP play a critical role in providing energy for immune cell metabolism and activation to fight against the infection and regulate the immune response. Remarkably, these metabolic and lipidomic changes offer great opportunities for the development of novel biomarkers and treatments for COVID-19 infection and pneumonia.

## Data availability

The data that support the findings of this study are available from the corresponding author, SP upon reasonable request.

## Supplemental data

This article contains supplemental data.

## Conflicts of interest

The authors declare that they have no conflicts of interest with the contents of this article.
