# Supplementary figures and images for: Lipidomic and metabolomic changes in community-acquired and COVID-19 pneumonia
Source: J Lipid Res. 2024 Aug 21;65(9):100622. doi: 10.1016/j.jlr.2024.100622 (PMC11422144; doi:10.1016/j.jlr.2024.100622)

## Supplementary Fig. 2

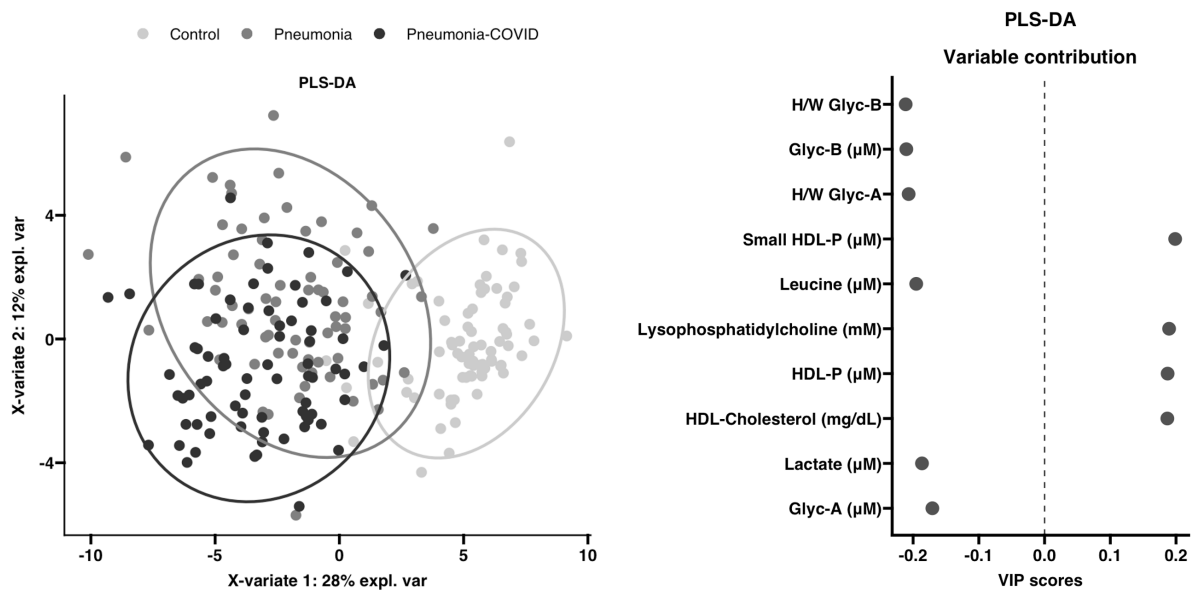

## Supplementary Fig. 2

[illegible]

Supplement: Supplementary Figure [file mmc1.pdf]
